# Transformer-based model for predicting length of stay in intensive care unit in sepsis patients

**DOI:** 10.3389/fmed.2024.1473533

**Published:** 2025-01-07

**Authors:** Jeesu Kim, Geun-Hyeong Kim, Jae-Woo Kim, Ka Hyun Kim, Jae-Young Maeng, Yong-Goo Shin, Seung Park

**Affiliations:** ^1^Medical Artificial Intelligence Center, Chungbuk National University Hospital, Cheongju, Republic of Korea; ^2^College of Medicine, Chungbuk National University, Cheongju, Republic of Korea; ^3^Department of Electronics and Information Engineering, Korea University, Sejong, Republic of Korea; ^4^Department of Biomedical Engineering, Chungbuk National University Hospital, Cheongju, Republic of Korea

**Keywords:** sepsis, intensive care unit, length of stay, sequential organ failure assessment, transformer, tabular data

## Abstract

**Introduction:**

Sepsis, a life-threatening condition with a high mortality rate, requires intensive care unit (ICU) admission. The increasing hospitalization rate for patients with sepsis has escalated medical costs due to the strain on ICU resources. Efficient management of ICU resources is critical to addressing this challenge.

**Methods:**

This study utilized the dataset collected from 521 patients with sepsis at Chungbuk National University Hospital between July 2020 and August 2023. A transformer-based deep learning model was developed to predict ICU length of stay (LOS). The model incorporated global and local input data analysis through classification and feature-wise tokens, based on sequential organ failure assessment (SOFA) criteria. Model performance was evaluated using four-fold cross-validation.

**Results:**

The proposed model achieved a mean absolute error (MAE) of 2.05 days for predicting ICU LOS. The result demonstrates the ability of the proposed model to provide accurate and reliable predictions.

**Discussion:**

The proposed model offers valuable insights for healthcare resource management by optimizing ICU resource allocation and potentially reducing medical expenses. These findings highlight the applicability of the proposed model to efficient healthcare cost management.

## 1 Introduction

Sepsis, a life-threatening condition, arises when the body’s response to infection induces widespread inflammation (1). This inflammatory response can damage multiple organ systems, leading to severe multi-organ failure (2). The rapid progression of sepsis can result in death without timely and appropriate treatment (3). Global guidelines often recommend intensive care for patients with sepsis (2). Despite advancements in medical technology, including early diagnostic methods, rapid antibiotic administration (4), and advanced supportive care such as mechanical ventilation and extracorporeal membrane oxygenation (5), sepsis continues to pose a significant healthcare challenge worldwide due to increasing mortality and morbidity rates (6–11).

Treating sepsis in the intensive care unit (ICU) incurs significantly higher costs than treating other diseases owing to the need for advanced life support measures, prolonged hospitalization, and the complexity of managing multi-organ failure (3, 12, 13). Recent statistics indicate that the annual cost of treating sepsis in the United States exceeds $24 billion, making it the most expensive treatment option for hospitals (13). This high cost is primarily driven by the length of stay (LOS) in the ICU because patients with sepsis often require prolonged intensive care. Sepsis accounts for 4.7–42.2% of global ICU utilization because ICU admission is recommended as an aggressive treatment regimen (14). Additionally, sepsis readmission rates are alarmingly high, with approximately 19% of survivors readmitted within 30 days, further escalating healthcare expenditures (15). Accurate prediction of ICU LOS for sepsis patients is crucial, as it enables healthcare facilities to optimize resource allocation, such as bed utilization, staffing, and equipment availability. By improving care efficiency, hospitals can reduce operational costs, enhance patient turnover rates, and ultimately contribute to cost savings for both healthcare providers and the broader system (16, 17).

.Efforts to predict ICU LOS have significantly advanced in recent years. In 2022, Wu et al. (18) demonstrated the utility of machine learning techniques by predicting ICU LOS (area under the receiver operating characteristic curve (AUROC) = 0.742) using gradient boosting decision trees (GBDTs). In the same year, Deng et al. (19) improved accuracy (AUC = 0.765) by utilizing temporal data and focusing on the changes in progression according to treatment stages using gated recurrent units (GRU) and long short-term memory (LSTM) networks. In 2023, the emphasis shifted to simpler, clinically interpretable models, such as linear regression models utilizing the sequential organ failure assessment (SOFA) score. Zangmo and Khwannimit (20) developed a model to classify sepsis patients with ICU LOS exceeding 3 days (AUC = 0.530), while Farimani et al. (21) proposed a model to predict ICU LOS in cardiac surgery patients (root mean square error (RMSE) = 5.181). Despite the advances, existing models have struggled to effectively capture the complex feature interactions inherent in structured data. GBDT emphasizes individual feature importance through splits (22), making it less effective at explicitly modeling complex interactions or high-dimensional relationships. On the other hand, GRU and LSTM models are optimized for processing sequential data, the models exhibit structural limitations in learning complex inter-variable relationships in structured datasets (19). Similarly, linear regression assumes linear relationships between variables and is, therefore, unable to capture nonlinear interactions (23). The limitations underscore the necessity for innovative methods capable of effectively learning complex and high-dimensional data structures. Hence, we present a transformer-based solution to address the limitations of structured data analysis by simultaneously capturing nonlinear feature interactions and learning global relationships through attention mechanisms.

Transformers have shown promise in structured data analysis by incorporating innovative mechanisms such as the classification (CLS) token, a functionality originally introduced in the bidirectional encoder representations from transformers (BERT) (24) in 2018. The CLS token serves as a global representation of the input sequence, summarizing overall patterns in structured data through self-attention, and allows Transformers to effectively capture global dependencies across features. In 2021, Models such as self-attention and intersample attention transformer (SAINT) (25) and feature tokenizer (FT)-Transformer (26) successfully leveraged CLS tokens, achieving performance improvements in tabular datasets. SAINT enables feature-to-feature and sample-to-sample interactions, while the FT-Transformer captures intricate inter-feature relationships and global patterns. However, while the CLS token excels at capturing global information, achieving a comprehensive analysis of attention mechanisms using CLS tokens can be challenging, as attention mechanisms may ignore important local feature details, particularly in datasets with complex interdependencies (27–29). The limitation of the CLS token underscores a persistent challenge in Transformer-based models when applied to highly intricate structured data.

This study focuses on predicting the ICU LOS for patients with sepsis using a transformer-based DL model applied to SOFA-based tabular data. The proposed model uses an attention mechanism and a skip-connected token process, integrating global information from a CLS token and local information from feature-wise tokens during the final classification. This approach adds to the growing body of work on applying DL techniques to tabular data in predicting ICU LOS for patients with sepsis.

## 2 Methods

### 2.1 Dataset information

#### 2.1.1 Study population

To develop the DL model, we constructed a dataset from patients treated for sepsis at Chungbuk National University Hospital (Cheong-Ju, Korea) between July 3, 2020 and August 3, 2023. The study, conducted following the principles of the Declaration of Helsinki, received approval from the Institutional Review Board of Chungbuk National University Hospital (IRB no. CBNUH 2021-02-034-001). Patient information was anonymized and de-identified prior to analysis.

**Figure 1:**
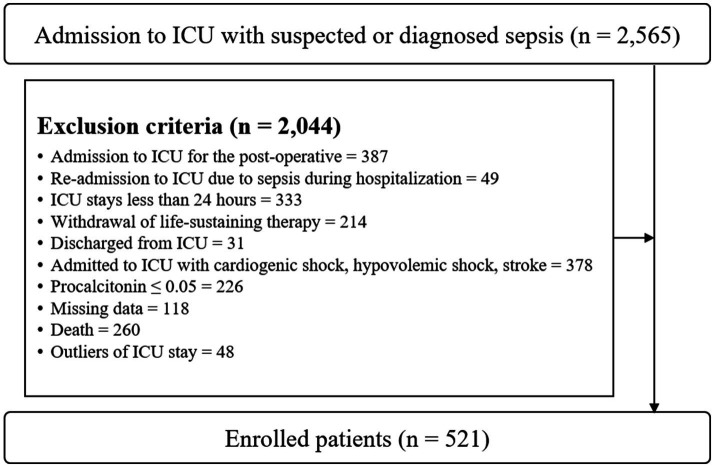
Flow diagram of the study inclusion and exclusion.

As shown in Figure 1, we initially identified patients meeting the Sepsis-3 guidelines for suspicion or diagnosis of sepsis, defined as a quick SOFA (qSOFA) score of ≥ 2. We sequentially excluded patients who met the following criteria: ICU admission post-surgery, readmission due to sepsis during treatment, ICU stays of less than 24 h, withdrawal of life-sustaining therapy, ICU discharge, admission with cardiogenic shock, hypovolemic shock, or acute stroke, procalcitonin level of ≤ 0.05, missing data, death, and ICU LOS outliers. This process resulted in a dataset comprising 521 patients.

We collected various clinical and SOFA-related features to construct a sepsis-specific ICU LOS prediction model. The features included: (1) Clinical features: age, sex, body mass index (BMI), lactate, atrial fibrillation (AF), systolic blood pressure(SBP), diastolic blood pressure (DBP), mean blood pressure (MBP), partial pressure of arterial oxygen and fraction of inspired oxygen ratio (PaO2/FiO2, P/F ratio), Glasgow Coma Scale (GCS)]. (2) SOFA-related features: vasopressor (VASO), mechanical ventilator (MV), 24-h urinary excretion (UR), platelets (Plt), serum total bilirubin (Bil), serum creatinine (Cr)]. (3) Target feature: ICU LOS.

Table 1 presents detailed statistical information on the features used in this study. Numerical features are described using means, standard deviations, and min-max ranges, while categorical features are reported as frequencies and percentages. The Pearson correlation coefficient for numerical features and point-biserial correlation for categorical features were calculated to determine their correlation with the target feature. *p*-values in Table 1 test the null hypothesis that the correlation coefficient is zero.

**Table 1:** Statistical information of features in our dataset.

Characteristics	Categories		Dataset (*n* = 521)	Correlation coefficient	*p*-value
Clinical features	Age	Years	69.19 ± 14.65 (19–95)	−0.09	0.05
	Sex	Female	239 (45.87%)	0.01	0.90
		Male	282 (54.13%)		
	BMI	kg/cm^2^	22.41 ± 4.18 (11.55–43.94)	−0.07	0.11
	LACTATE	mmol/L	2.58 ± 2.56 (0–29)	0.16	< 0.01
	AF	yes	86 (16.51%)	0.06	0.19
		no	435 (83.49%)		
	SBP	mmHg	89.67 ± 19.59 (33–176)	−0.08	0.08
	DBP	mmHg	49.31 ± 11.46 (17–90)	−0.09	0.04
	MBP	mmHg	60.28 ± 11.83 (24–104)	−0.10	0.03
	PF	-	290.69 ± 152.05 (16–943)	−0.31	< 0.01
	GCS	-	11.08 ± 3.55 (3–15)	−0.42	< 0.01
SOFA-related features	VASO	Yes	298 (57.2%)	0.11	0.02
		No	223 (42.8%)		
	MV	Yes	171 (32.82%)	0.43	< 0.01
		No	350 (67.18%)		
	UR	cm^3^	1803.52 ± 1332.96 (0–1,250)	−0.19	< 0.01
	Plt	×10^3^/μl	154.16 ± 96.02 (4–657)	−0.06	0.19
	Bil	mg/dl	1.31 ± 2.09 (0.09–22.58)	−0.02	0.72
	Cr	mg/dl	2.14 ± 2.20 (0.11–16.60)	0.07	0.13
Target feature	ICU LOS	days	5.24 ± 3.57 (1.01–16.47)		

SOFA, sequential organ failure assessment; BMI, body mass index; AF, atrial fibrillation; SBP, systolic blood pressure; DBP, diastolic blood pressure; MBP, mean blood pressure; P/F ratio, partial pressure of arterial oxygen and a fraction of inspired oxygen ratio (PaO_2_/FiO_2_); GCS, Glasgow Coma Scale; ICU LOS, length of stay in intensive care unit; VASO, vasopressor; MV, mechanical ventilator; UR, 24 h urinary excretion; Plt, platelets; Bil, serum total bilirubin; Cr, serum creatinine.

#### 2.1.2 Data preprocessing

**Figure 2:**
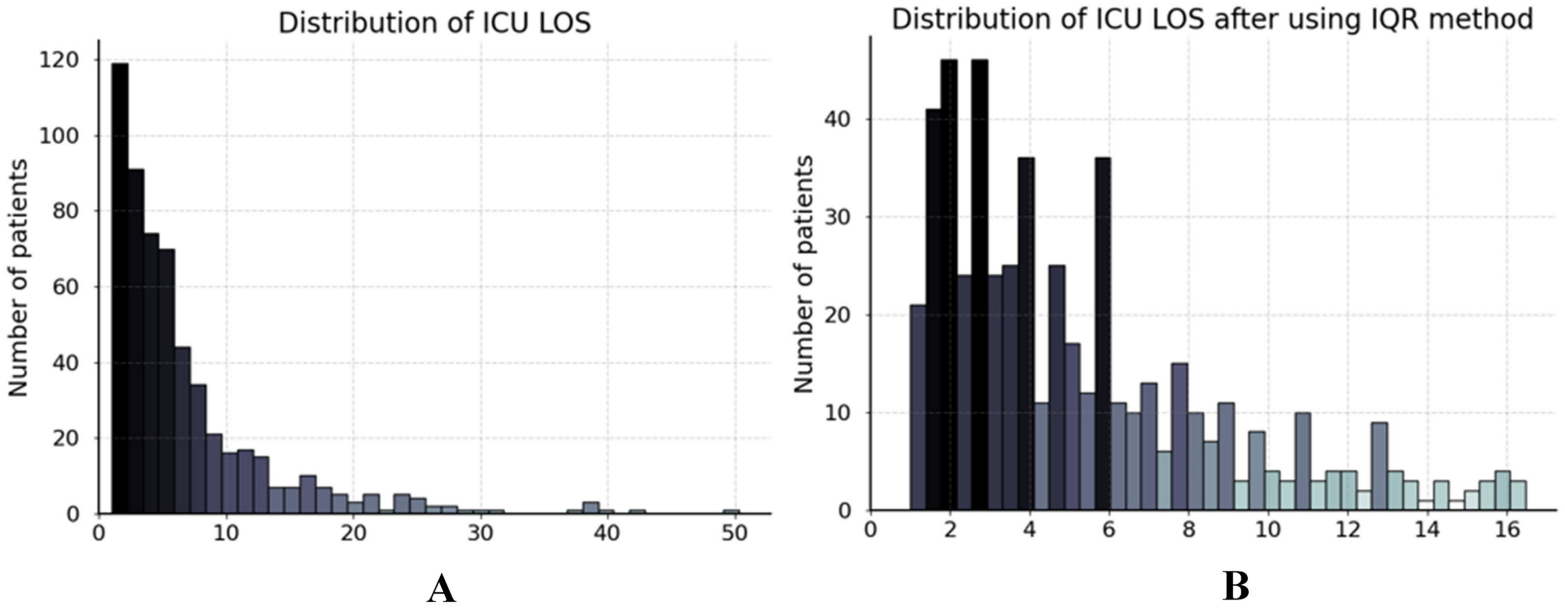
Distribution of ICU LOS in dataset. **(A)** The ICU LOS before data preprocessing and **(B)** the ICU LOS after handling outliers using the IQR method.

As depicted in Figure 2A, the ICU LOS distribution in our dataset exhibited a pronounced positive skew, with a concentration of values at the lower end and a long tail extending towards higher values. This necessitated the removal of outliers prior to analysis. The interquartile range (IQR) method was employed to handle outliers in ICU LOS (30). The IQR method effectively retains most data points within a reasonable range, excluding outliers that could potentially distort the analysis (31). Specifically, the IQR is the range between the first quartile (Q1) and third quartile (Q3) of the data, with outliers defined as points below Q1–1.5IQR or above Q3 + 1.5IQR (32). Our study identified patients with an ICU LOS > 16.52 days as outliers, excluding 48 patients as shown in Figure 2B. Furthermore, we standardized the dataset to ensure that all features contributed equally to the analysis and to prevent any single feature from disproportionately influencing the results due to scale differences. This procedure was applied exclusively to numerical features. The standardization formula is defined in Equation 1 as follows:


(1) $$Z=\frac{\left(X-\bar{X}\right)}{s}\text{,}$$

where $\bar{X}$ represents the mean and s denotes the standard deviation (33).

### 2.2 Model architecture

**Figure 3:**
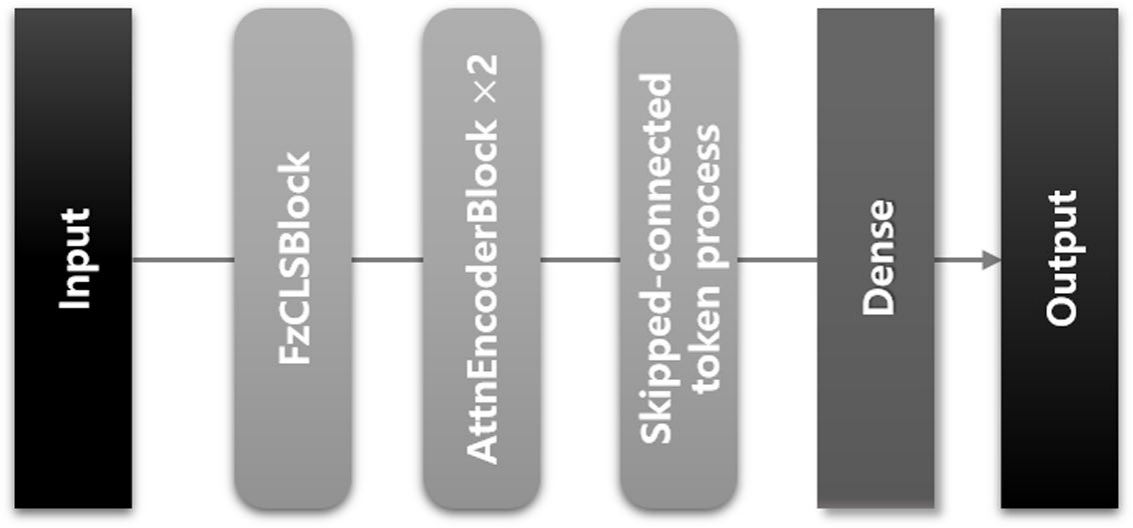
Architecture of the proposed model for predicting ICU LOS in patients with sepsis.

**Figure 4:**
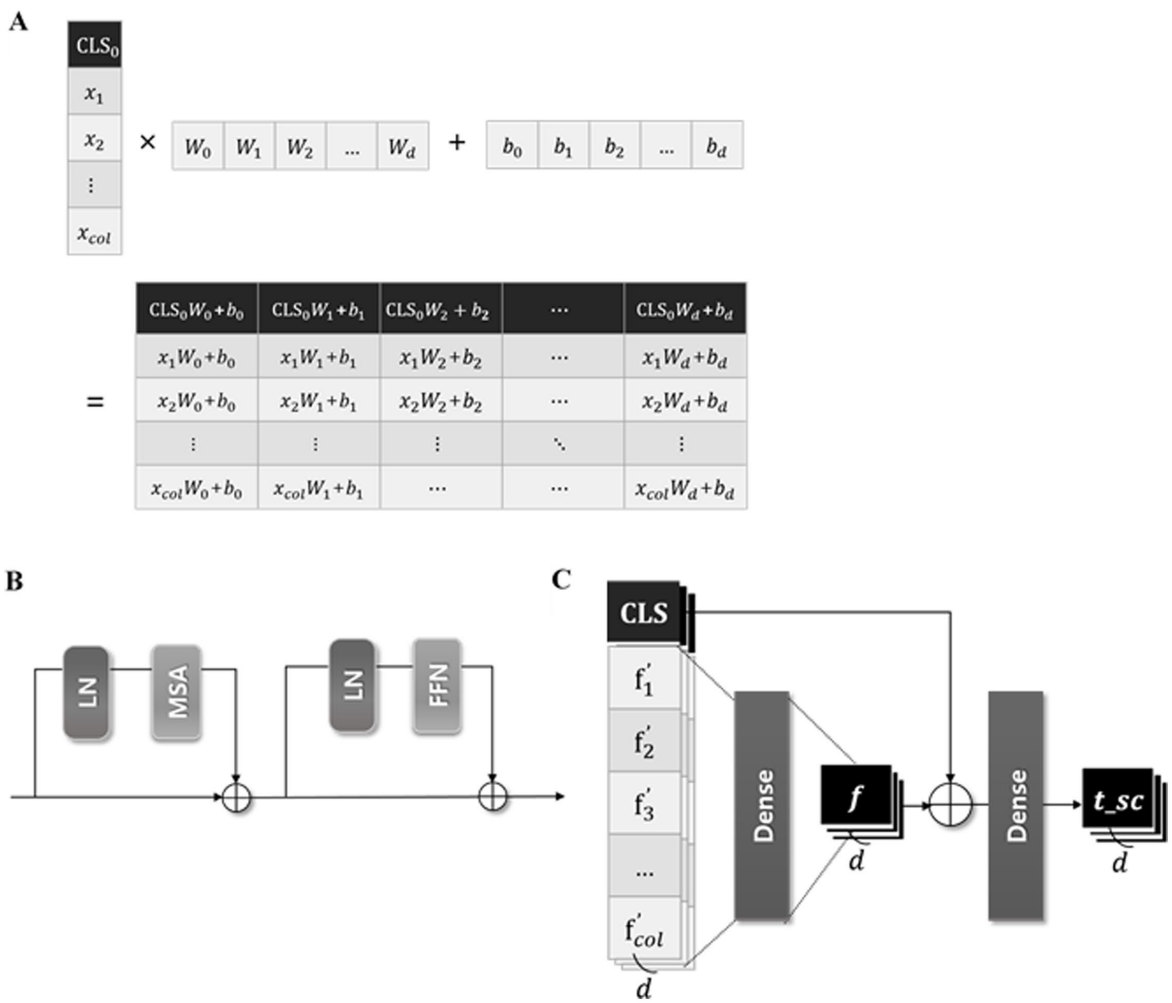
Three modules of the proposed model. **(A)** The illustration of the process concatenating CLS token in FzCLSBlock, **(B)** schematic diagram of the module of multi-head self-attention, **(C)** process of global and local information analysis.

We developed a transformer-based DL model using a CLS token to predict the ICU LOS. The architecture of the proposed model is depicted in Figure 3; it consists of three modules as shown in Figure 4.

#### 2.2.1 Module of concatenating CLS tokens

The input data $x\in {\mathbb{R}}^{\mathrm{col}}$ , where col represents the number of input features, is batch normalized before entering the “fzCLSBlock.” As represented in Figure 4A, x in the fzCLSBlock is concatenated with a trainable CLS token ${CLS}_{0}$ $\in {\mathbb{R}}^{1}$ , which is zero-initialized to ensure stable training (24). The CLS token is the first special token of every sequence and is widely used as an aggregate sequence representation for classification tasks (26). The concatenated vectors are embedded through a dense layer to achieve a representative embedding of the input data and capture the complex relationships. This process is expressed in Equation 2 as follows:


(2) $$z=\mathrm{Dense}\left({CLS}_{0}∥x\right)\in {\mathbb{R}}^{\left(\mathrm{col}+1\right)\times d}\text{,}$$

where *d* represents the embedding dimension and ∥ denotes the concatenate function.

#### 2.2.2 Module of multi-head self-attention

Inspired by networks in several studies using transformers (25, 34–36), we employed the self-attention mechanism of the transformer encoder. The self-attention mechanism calculates model weights to assess the relevance of each feature and captures interactions between features or instances. Recent research have demonstrated superior prediction accuracy by incorporating self-attention mechanisms in new networks such as TabNet (35) and FT-transformers (26). These findings suggest the efficacy of self-attention mechanisms for analyzing tabular datasets.

The projected vector $z\in {\mathbb{R}}^{\left(\mathrm{col}+1\right)\times d}$ (as defined above) is analyzed in the “AttnEncoderBlock” illustrated in Figure 4B. The input vector z is linearly transformed into query (Q), key (K), and value (V) matrices within the single attention head of multihead self-attention (MSA) (37). The attention weight is calculated by taking the dot product of Q and K, normalizing it by the square root of the dimension of K, and applying a softmax function. After that, the attention head outputs the dot product of the attention score and V, which are computed in parallel five times. Besides the MSA, the AttnEncoderBlock includes a fully connected feedforward network (FFN) composed of two linear transformations with a rectified linear unit (ReLU) activation in between (38).

#### 2.2.3 Module of analyzing global and local information

Previous research indicates that CLS tokens often fail to adequately capture the semantic content of the input because they focus more on global information than local and low-level features (39). We designed a skip-connected token process, which comprehensively analyzes global and local data information, to address this issue. A skip-connected token process addes token values representing both global and local information.

As presented in Figure 4C, the output of the AttnEncoderBlock is batch normalized and divided into the $CLS\in {\mathbb{R}}^{1\times d}$ token, summarizing all features, and the feature-wise token $f^{\prime }\in {\mathbb{R}}^{\mathrm{col}\times d}$ , maintaining the unique information of each feature. The $f^{\prime }$ , containing local information, passes through a dense layer to convert the local information into more abstract and high-level features. This layer captures complex dependencies and correlations between local features by identifying the interactions between various feature dimensions and learning appropriate weights. Additionally, the CLS token, containing global information, is added to f , enabling a comprehensive analysis of global and local information. These computations can be expressed in Equations 3 and 4 as follows:


(3) $$f=\mathrm{Dense}\left(\mathrm{Flatten}\left(f^{\prime }\right)\right)\in {\mathbb{R}}^{d}\text{,}$$

(4) $$t_{sc}=\mathrm{Dense}\left(CLS+f\right)\in {\mathbb{R}}^{d}\text{.}$$

The $t_{sc}$ token is used for the final prediction of the proposed model, predicting the ICU LOS via a dense layer with one unit.

### 2.3 Implementation details

The proposed model was implemented using Python 3.9 on a workstation with an 11th Gen Intel(R) Core(TM) i7-11700K processor at 3.60 GHz and 64 GB of RAM. We applied exponential decay to control the learning rate during training, gradually reducing it to ensure stable convergence. The proposed model was configured with a batch size of 32 and the Adam optimizer at a learning rate of 1e-3. Learning rate decay was applied every 10 steps at a rate of 0.96. Furthermore, we compared the prediction accuracy of the proposed model with that of conventional ML and DL models. Hyperparameters for random forest (RF) (40), extreme gradient boosting (XGBoost) (40), support vector regression (SVR) (41), multiple linear regression (MLR) (42), and TabNet (35) were set to their respective default values.

### 2.4 Model performance evaluation

We conducted a four-fold cross-validation to verify the reliability and consistency of the predictions of the proposed model. Twenty percent of the dataset was allocated for testing, while the remaining dataset was divided into four folds. Each iteration of the four-fold validation consisted of one fold used for validation and the remaining folds used for training. We adopted the following three key metrics to quantitatively evaluate the performance of the proposed model because it performed a regression task: coefficient of determination (R^2^), mean absolute error (MAE), and root mean square error (RMSE). Detailed descriptions of each metric are as follows:

The R^2^ value measures the proportion of variance in the dependent feature that can be predicted from the independent features. The R^2^ value ranges from 0 to 1, where 0 indicates that the model does not explain the variability in the response data around its mean, and 1 indicates that the model explains all the variability of the response data around its mean (43). R^2^ value for an ideal model is close to 1 and is computed using Equation 5 as follows:


(5) $$R^{2}=1-\frac{\sum_{i=1}^{n}{\left(y_{i}-{\hat{y}}_{i}\right)}^{2}}{\sum_{i=1}^{n}{\left(y_{i}-\bar{y}\right)}^{2}}\text{,}$$

where n denotes the number of patients, $y_{i}$ corresponds to the observed value, ${\hat{y}}_{i}$ represents the predicted value, and $\bar{y}$ is the average ICU LOS. The R^2^ metric is crucial as it directly correlates with the proportion of the total variation in the target feature explained by the model. A high R^2^ indicates that the model captures a significant portion of the variance, vital for predictive accuracy (44).

The MAE represents the average absolute difference between the predicted and observed values of the model (45). It provides a straightforward and interpretable measure of the average prediction error (46). Ideally, the MAE value approaches zero and is computed using Equation 6 as follows:


(6) $$MAE=\frac{1}{n}\overset{n}{\underset{i=1}{\sum }}|y_{i}-{\hat{y}}_{i}|\text{,}$$

where *n* represents the number of data points used for model testing, ${\hat{y}}_{i}$ corresponds to the value predicted by the model for the i -th sample, and $y_{i}$ denotes the corresponding observed value (47). The MAE is advantageous due to its reduced sensitivity to outliers compared to metrics such as RMSE, making it a more reliable indicator of the average performance of a model, particularly when handling datasets with noisy or extreme values (48).

The RMSE is the square root of the average squared difference between the predicted and actual observations. It is widely used due to its ability to penalize larger errors more heavily than MAE, highlighting significant deviations (48). The formula for RMSE is calculated using Equation 7:


(7) $$MSE=\sqrt{\left(\frac{1}{n}\right)\overset{n}{\underset{i=1}{\sum }}{\left(y_{i}-{\hat{y}}_{i}\right)}^{2}}\text{.}$$

This metric provides an aggregate measure of model accuracy, encompassing both bias and variance components of error. RMSE is valuable in applications where larger errors are more significant and must be minimized. Its sensitivity to large errors makes it essential for ensuring robustness and precision (49).

## 3 Results

### 3.1 Model performance comparison

We conducted a performance comparison of the proposed model using a four-fold cross-validation of the datasets. In Table 2, the proposed model demonstrated promising predictive performance, achieving an average R^2^, MAE, and RMSE of 0.29 ± 0.01, 2.05 ± 0.03, and 2.72 ± 0.02, respectively. The average R^2^ indicates the proposed model could explain approximately 29% of the variability in ICU LOS. Notably, the R^2^, MAE and RMSE values showed minimal variation across folds, demonstrating the stability.

**Table 2:** Performance of the proposed model evaluated using four-fold cross-validation.

Four-fold cross-validation	CBNUH (521 patients)		
	R^2^	MAE	RMSE
Fold1	0.30	2.03	2.69
Fold2	0.28	2.06	2.73
Fold3	0.29	2.01	2.71
Fold4	0.27	2.08	2.74
Average	0.29 ± 0.01	2.05 ± 0.03	2.72 ± 0.02

**Figure 5:**
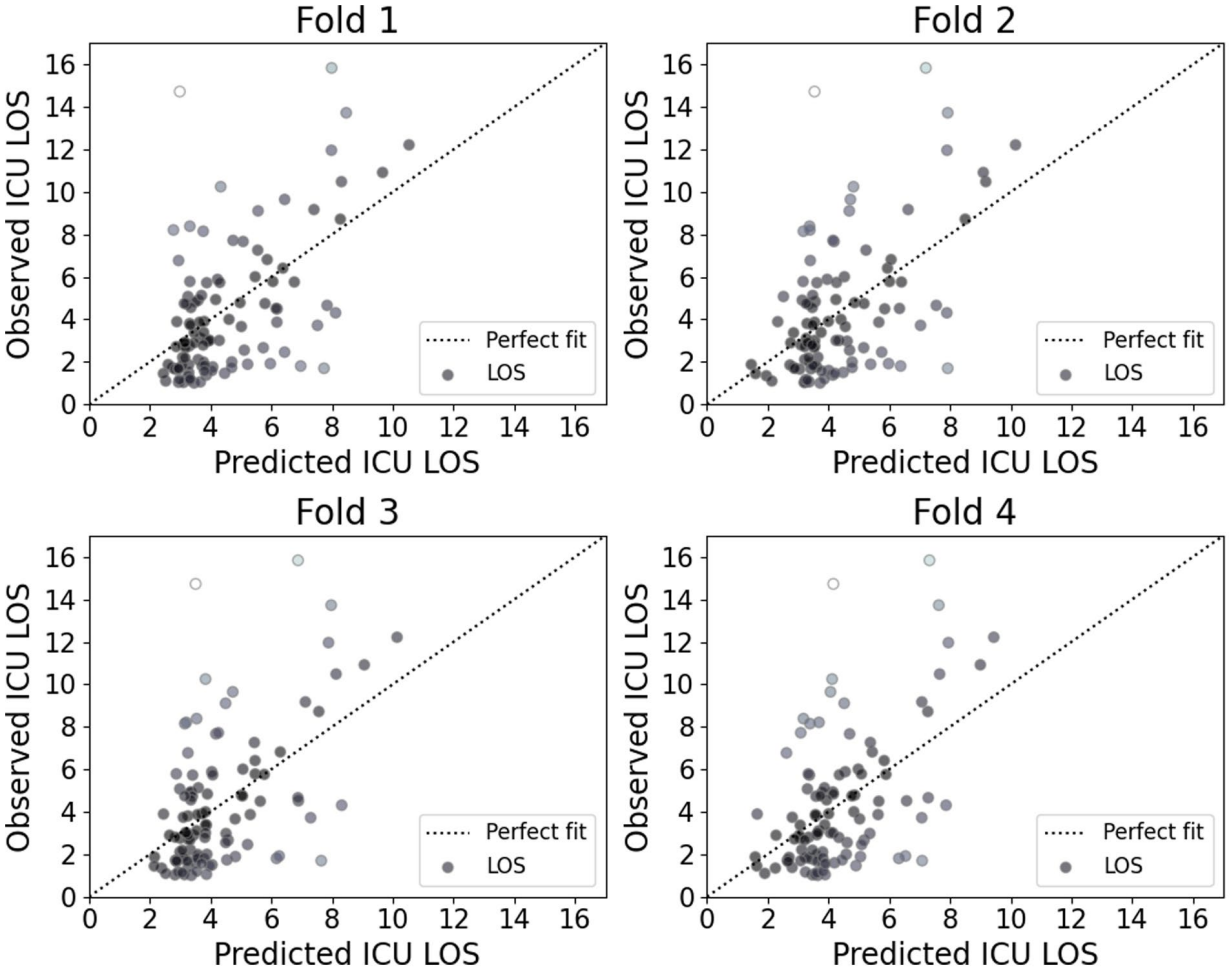
Calibration plot of each fold model. ICU, Intensive care unit; LOS, length of stay.

Figure 5 presents the calibration plot for each model, illustrating the agreement between predicted and observed ICU LOS. Darker points in these plots represent a better fit with actual values. The plots indicate that while the proposed model accurately predicts shorter ICU stays, it exhibits noticeable deviations for longer stays. This finding suggests that the proposed model demonstrated strong performance for shorter ICU stays; however, it may require further refinement to improve accuracy for longer stays, which are often associated with more complex and variable patient conditions.

Additionally, we compared the performance of the proposed model with conventional ML and DL models, as shown in Table 3. The proposed model leveraged the skip-connected token process to enhance its predictive power by capturing interactions within tabular data. Comparisons were made with other DL models using MSA, such as TabNet and FT-Transformer, as well as traditional models known for their strong performance on tabular data, including RF, XGBoost, and MLR. The proposed model demonstrated superior performance compared to the other models, indicating that it provides more accurate predictions.

**Table 3:** Comparison of conventional model performance.

Model	CBNUH		
	R^2^	MAE	RMSE
RF	0.09 ± 0.01	2.25 ± 0.01	3.07 ± 0.01
XGBoost	0.10 ± 0.02	2.23 ± 0.02	3.05 ± 0.04
MLR	0.22 ± 0.00	2.13 ± 0.01	2.84 ± 0.01
TabNet	−0.39 ± 0.21	2.77 ± 0.17	3.79 ± 0.29
FT-Transformer	0.26 ± 0.03	2.12 ± 0.02	2.76 ± 0.05
Proposed model	0.29 ± 0.01	2.05 ± 0.03	2.72 ± 0.02

### 3.2 Ablation study

We conducted an ablation study to evaluate the effectiveness of the proposed skip-connected token process. This study compared the information delivered to the ICU LOS output layer by altering specific components. The performance of models was compared across three categories: models that used only local information analysis, only global information analysis, or a combination of both. Detailed configurations and corresponding performance indicators are provided in Table 4.

**Table 4:** Ablation study on the proposed model.

Method	R^2^	MAE	RMSE
Local information analysis	0.22 ± 0.02	2.11 ± 0.06	2.84 ± 0.04
Global information analysis	0.15 ± 0.02	2.27 ± 0.04	2.97 ± 0.03
Local and Global information analysis	0.29 ± 0.01	2.05 ± 0.03	2.72 ± 0.02

The first model, which utilized only global information analysis, demonstrated poor performance. In contrast, the second model, relying solely on local information analysis, exhibited improved results. Notably, the proposed model, integrating global and local information analysis, achieved an R^2^, MAE, and RMSE of 0.29 ± 0.01, 2.05 ± 0.03, and 2.72 ± 0.02, respectively, outperforming the other two models.

These results indicate that the proposed model, which uses skip-connected token process, has the highest explanatory power and lowest prediction error, demonstrating a significant enhancement in overall model performance. This underscores the necessity of skip-connected token process in integrating local and global information for improved predictions.

### 3.3 Model interpretation

**Figure 6:**
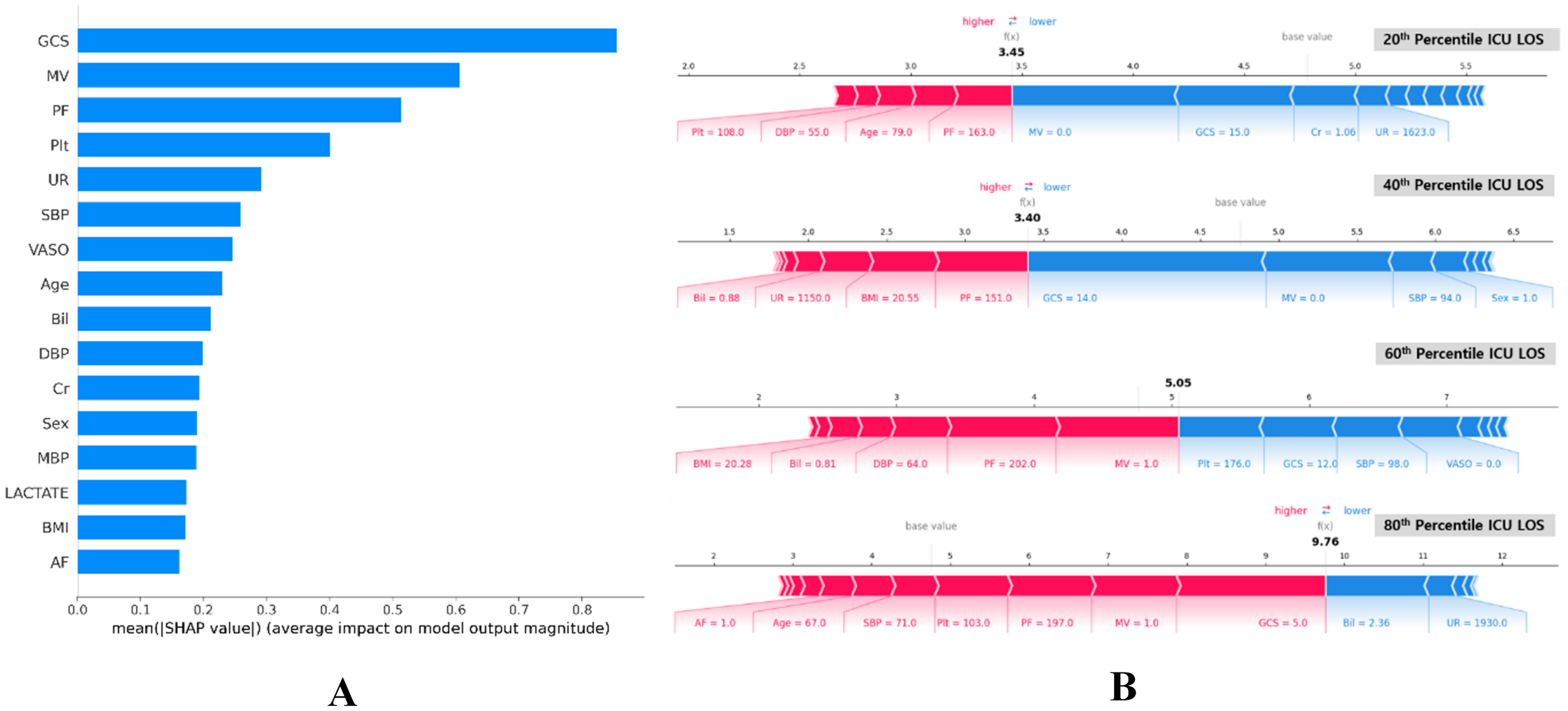
SHAP importance of features for predicting ICU LOS. **(A)** Summary plot of SHAP feature importance, represented by the mean absolute Shapley values. The plot illustrates the significance of each covariate in the final predictive model. **(B)** SHAP force plots for data instances with predicted ICU LOS at the 80^th^, 60^th^, 40^th^, and 20^th^ percentiles (bottom). These plots provide an explanation for individual predictions made by the model. Note: the base value of 4.77 days is consistent across all plots. P/F, partial pressure of arterial oxygen and fraction of inspired oxygen ratio (PaO_2_/FiO_2_); GCS, Glasgow Coma Scale; AF, atrial fibrillation; Cr, serum creatinine; VASO, vasopressor; Plt, platelets; MV, mechanical ventilator; UR, 24 h urinary excretion; Bil, serum total bilirubin; DBP, diastolic blood pressure; BMI, body mass index; SBP, systolic blood pressure; MBP, mean blood pressure; ICU LOS, length of stay in intensive care unit.

We employed Shapley additive descriptions (SHAP) to assess the impact of each feature on the model predictions. Figure 6A displays the mean absolute SHAP values for each feature, highlighting their importance in the model predictions. The top three most influential features were GCS, MV and PF. This ranking elucidates the primary factors that drive the predictions of the proposed model, offering valuable insights into which features most significantly affect ICU length of stay predictions.

Figure 6B presents the SHAP summary plots for four different percentiles (20th, 40th, 60th, and 80th) of ICU LOS. These plots visualize the SHAP values for individual predictions, indicating how each feature affects the predicted ICU LOS. The color scale shows the direction of the impact, with red and blue indicating an increase and decrease in the predicted LOS, respectively.

Sequentially across percentiles, the high GCS score and application of MV reduced predicted LOS, primarily at the 20th and 40th percentiles of ICU LOS (see blue section). Conversely, the low PF ratio increased LOS. The GCS represents a level of consciousness rating of 3–15 that assesses neurological status, with a lower score indicating worse status (50). The MV and PF are important indicators of respiratory function, reflecting the need for mechanical ventilation and the oxygen exchange capacity of the lungs, respectively (49). The impact of MV application and low PF was also evident at the 60th percentile, significantly increasing ICU LOS. On the other hand, an average level of Plt indicates a properly functioning coagulation system and reduces ICU LOS. The low GCS score, high Bil level, and MV application played a significant role in 80th percentile ICU LOS, with severe GCS score significantly increasing expected ICU LOS. Bil is another important predictor of ICU LOS, with elevated levels indicating liver dysfunction or hemolysis. However, contrarily in our study, elevated Bil was shown to reduce ICU LOS.

## 4 Discussion

This study demonstrated that the transformer-based DL model outperformed traditional ML and DL models in predicting ICU LOS for patients with sepsis using SOFA-based tabular data. The proposed model, leveraging a skip-connected token process to integrate global and local information, achieved an average R^2^, MAE, and RMSE of 0.29, 2.05 days, and 2.72 days, respectively. Reliable predictions of ICU LOS are clinically and operationally impactful, as they enable better resource allocation and improve patient outcomes, particularly for critical conditions like sepsis (51, 52). The proposed model builds on these insights by providing an efficient tool that uses limited SOFA-based data to achieve practical predictions.

The strengths of the proposed model are manifold: First, the input features are based on the SOFA criteria, widely used in ICUs to assess organ dysfunction severity in critically ill patients. The model requires only 16 SOFA-related clinical features collected within 24 h of ICU admission, making it a convenient tool for predicting ICU LOS in patients with sepsis due to the accessibility of SOFA criteria data. Second, the proposed model was designed to work with tabular data, the most common structured data format, which requires less computational power than other data types and does not necessitate high-end hardware. Third, the model effectively captures comprehensive information from the features utilizing CLS and feature-wise tokens, analyzing global and local information. The proposed model employs MSA to capture global interactions between features, further analyze local information through dense layers and then integrates both in the final prediction to enhance performance.

Furthermore, the proposed model was interpreted using SHAP, providing valuable insights into the relative importance of various features in predicting ICU LOS. The top three influential features in this study were GCS, MV, and PF. The GCS score, underscored for its critical role in assessing neurological status, showed a positive correlation with ICU LOS. This finding is consistent with a previous study (53), and highlights the importance of GCS as the most significant predictor. Similarly, MV and PF are respiratory indices associated with ICU LOS prediction in this study. The results in our study are consistent with previous studies showing that MV use and lower PF increased ICU LOS (53, 54). Conversely, this study found that elevated bilirubin levels were associated with a shorter ICU LOS, which contrasts with a previous study where higher bilirubin levels prolonged the length of hospital stay (55). The correlation of these factors indicates that these may assist in determining ICU LOS.

However, this study has several limitations. The dataset was derived from a single institution, potentially limiting the generalizability of the findings. Future research should aim to validate the proposed model across diverse healthcare settings and larger multicenter datasets. Additionally, while the transformer-based model outperformed others in predicting ICU LOS, it showed an opportunity for improvement, particularly in predicting stays longer than 8 days. This result suggests the need for additional data on longer durations to improve the prediction of extended ICU LOS in real medical scenarios.

## 5 Conclusion

We developed a transformer-based DL model to predict ICU LOS in patients with sepsis using data collected within the first 24 h of ICU admission. The proposed model achieved an MAE of 2.05 days. The proposed model effectively captures complex feature interactions by integrating global and local information through a novel skip-connected token process. Additionally, the proposed model utilizes a set of SOFA-related features that are widely used to assess the severity of organ dysfunction in clinical practice. Such an approach ensures simplicity of data collection and wide applicability, making the proposed model practical for use in a variety of healthcare settings.

## Data Availability

The raw data supporting the conclusions of this article will be made available by the authors, without undue reservation.
